# Fomite advice for scabies: a review of online resources

**DOI:** 10.1093/skinhd/vzae014

**Published:** 2025-02-27

**Authors:** Kemi Fabusiwa, Eglantine Lebas, Kar Hung Kuet, Ananth K Nalabanda, Rebecca L McCarthy, Stephen L Walker

**Affiliations:** Department of Dermatology, University College London Hospitals NHS Foundation Trust, London, UK; St John’s Institute of Dermatology, Guy’s and St Thomas’ Hospital NHS Foundation Trust, London, UK; Department of Dermatology, Sheffield Teaching Hospitals NHS Foundation Trust, Sheffield, UK; Centre for Primary Care and Public Health, Barts and The London School of Medicine and Dentistry, Queen Mary University of London, London, UK; Department of Dermatology, Imperial College Healthcare NHS Trust, London, UK; Blizard Institute, Faculty of Medicine and Dentistry, Queen Mary University of London, London, UK; Department of Dermatology, University College London Hospitals NHS Foundation Trust, London, UK; Hospital for Tropical Diseases, University College London Hospitals NHS Foundation Trust, London, UK; Faculty of Infectious and Tropical Diseases, London School of Hygiene and Tropical Medicine, London, UK

## Abstract

Scabies is a significant global public health issue that affects 200 million people worldwide. We reviewed English language, publicly available online resources concerning the management of scabies for guidance on fomites. Information was collected from 10 resources in March 2024. We believe the quality of information used to advise individuals about appropriate decontamination of fomites should be improved.

Dear Editor, Scabies is a significant global public health issue that affects 200 million people worldwide.^1^ The *Sarcoptes scabiei* var. *hominis* mite causes an intensely pruritic, characteristic rash that results in significant morbidity and secondary bacterial infection.^2^ Scabies primarily affects children, older and immunocompromised individuals, and people residing in resource-limited communities.^3^ There is no evidence of drug resistance, but treatment failure is estimated to occur in 30% of cases, due to adherence, topical therapy application technique, and reinfestation from an affected individual or fomites.^4^

Prolonged skin-to-skin contact remains the primary transmission route;^5^ however, the ability of mites to survive off the host for 24–48 h allows transmission via fomites.^6^ Fomites are inanimate objects or materials capable of serving as intermediaries for transmission.^7^ Appropriate, comprehensible advice for affected individuals and their contacts is essential for the optimal management of scabies, and this includes guidance on fomites.

We reviewed English language, publicly available online resources concerning the management of scabies, specifically focusing on guidelines published by medical professionals’ associations, governmental agencies and public health organizations. Information was collected from 10 resources in March 2024. Table 1 shows a summary of recommendations.

**Table 1:** Recommendations for the management of fomites by health information organizations

Action recommended	Organization responsible for scabies advice									
	AAD^8^	ACD^9^	BAD^10^	BASHH^11^	CDC^12^	EADV^13^	NHS^14^	NICE^15^	PCDS^16^	WHO^17^
Temperature of washing	‘Hottest water possible’	Hot water	‘High’	60 °C	‘Hot’	≥50 °C	≥50 °C	≥60 °C	≥50 °C	‘Hot water’
Duration in sealed bag	≥1 week	1 week	≥1 week	≥72 h	≥72 h	≥1 week	≥72 h	≥72 h	≥72 h	1 week
Dry clean	Yes	Yes	Yes	Yes	Yes	Yes	—	Yes	Yes	Yes
Tumble drying	Yes	Yes	—	Yes	—	—	—		Yes	Yes
Freeze	—	—	Yes	—	—	—	—	—	Yes	—
Ironing	—	Yes	—	—	—	—	—	—	Yes	—
Household cleaning	Vacuum entire home	—	—	—	—	—	—	—	—	Clean and vacuum or sweep
Household fumigation	—	No	—	—	No	—	—	—	No	—

AAD, American Academy of Dermatology; ACD, Australian College of Dermatology; BAD, British Association of Dermatologists; BASHH, British Association for Sexual Health and HIV; CDC, Centers for Disease Control; EADV, European Association of Dermatology and Venereology; NHS, National Health Service; NICE, National Institute for Health and Care Excellence; PCDS, Primary Care Dermatology Society; WHO, World Health Organization.

There was unified consensus that washing fomites with hot water should be employed. Five of 10 resources recommended water temperatures of 50 °C or higher. The remaining five did not specify a minimum temperature and instead used nonspecific terms like ‘hot’ or ‘high’ for washing temperature The fomites explicitly referred to included bedding, clothing and towels.

All 10 resources discussed placing items in a sealed plastic bag to isolate fomites from human contact where items are not amenable to washing or where power or domestic appliances are not available. Five of 10 resources recommended an isolation period of at least 72 h with the remainder recommending up to 1 week.

Dry cleaning was recommended by 9 of 10 resources, but specifications about what constitutes effective dry cleaning were not provided. Tumble drying was included in recommendations by five resources, and freezing and ironing by two. However, all lacked crucial details for those undertaking the tasks. The sources did not specify the duration needed for each method or the precise temperature extremes necessary to eliminate the mites and their by-products.

Two sources mentioned vacuuming or cleaning the environment, but, importantly, three resources explicitly stated that household fumigation is unnecessary. Interestingly, none of the reviewed sources addressed routine household cleaning practices.

Resources focused on the management of clothing and bedding while often omitting to mention towels. Other fomites, such as children’s toys, furnishing and car seats, were not addressed, although there is evidence they might contribute to treatment failure.^4^ The resources do not provide advice about household items that cannot easily be washed, tumble-dried, frozen or sealed in a bag, or reassurance when interventions are unnecessary. Such advice would reduce anxiety concerning which items may facilitate scabies transmission and limit the financial burden of unnecessarily replacing expensive items.

Our review is limited by its scope. We reviewed guidance from organizations based in high income settings which may not be applicable to individuals who lack access to water and sanitation facilities, electricity and domestic appliances.

We have identified inconsistencies and gaps in currently available guidance on fomites that potentially cause confusion. Bernigaud and colleagues highlighted the variation in decontamination protocols for fomites in 2020.^18^ Using a porcine model, they exposed *S. scabiei scabiei* to 89 environmental conditions through commonly recommended decontamination practices and assessed mite mortality and ova viability. The effect of exposure of mites to different temperatures for different durations was assessed. Temperatures of 50 °C for 10 min or −10 °C for 5 h were sufficient to kill all mites. The duration of isolation required to ensure mite death in sealed plastic bags under different climatic conditions was defined. Interestingly, detergent or ozone laundry had little effect on mites. Bernigaud and colleagues used the results of these studies to inform strategies for fomite decontamination illustrated in a useful infographic that should inform resources for healthcare professionals and affected individuals. Many of the resources we reviewed could give more detailed, specific and consistent advice by incorporating the findings and recommendations of Bernigaud *et al*.

To effectively address this global public health challenge, we advocate for improved information on *S. scabiei* fomite decontamination for affected individuals and healthcare providers. Further research is needed to clarify the role of fomites in persistent infestation despite adequate treatment and outbreaks in ‘closed’ settings, such as hostels, hospitals, long-term care facilities and prisons.

## Data Availability

The data underlying this article are available in the article.
